# Targeting Toll-like Receptor (TLR) Pathways in Inflammatory Arthritis: Two Better Than One?

**DOI:** 10.3390/biom11091291

**Published:** 2021-08-30

**Authors:** Sandra Santos-Sierra

**Affiliations:** Institute of Biochemical Pharmacology, Medical University of Innsbruck, Peter Mayr Str. 1, 6020 Innsbruck, Austria; Sandra.santos@i-med.ac.at

**Keywords:** Toll-like receptor, inhibitors, inflammation, arthritis, SLE, SpA, antagonists, DAMPs

## Abstract

Inflammatory arthritis is a cluster of diseases caused by unregulated activity of the immune system. The lost homeostasis is followed by the immune attack of one’s self, what damages healthy cells and tissues and leads to chronic inflammation of various tissues and organs (e.g., joints, lungs, heart, eyes). Different medications to control the excessive immune response are in use, however, drug resistances, flare-reactions and adverse effects to the current therapies are common in the affected patients. Thus, it is essential to broaden the spectrum of alternative treatments and to develop disease-modifying drugs. In the last 20 years, the involvement of the innate immune receptors TLRs in inflammatory arthritis has been widely investigated and targeting either the receptor itself or the proteins in the downstream signalling cascades has emerged as a promising therapeutic strategy. Yet, concerns about the use of pharmacological agents that inhibit TLR activity and may leave the host unprotected against invading pathogens and toxicity issues amid inhibition of downstream kinases crucial in various cellular functions have arisen. This review summarizes the existing knowledge on the role of TLRs in inflammatory arthritis; in addition, the likely druggable related targets and the developed inhibitors, and discusses the pros and cons of their potential clinical use.

## 1. Toll-like Receptor Signalling and Innate Immunity

**Figure 1:**
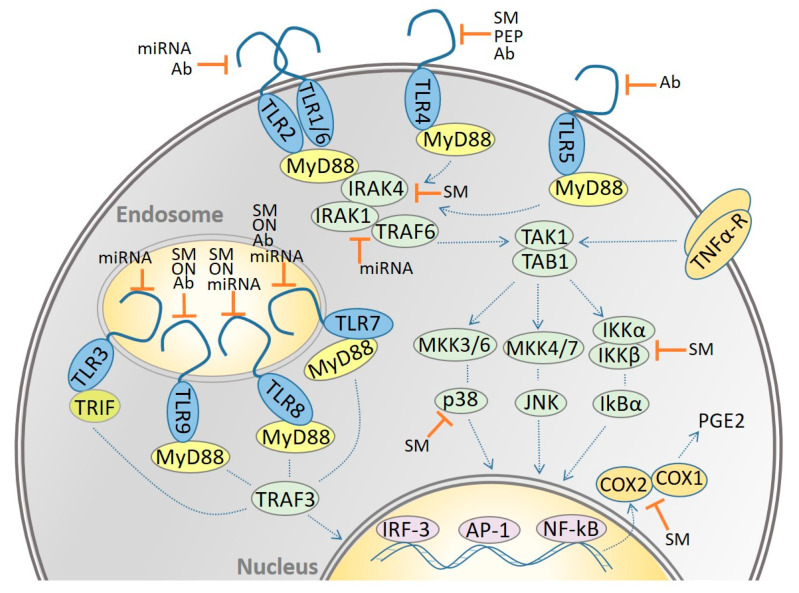
Constituents of the TLR pathways that have been targeted in inflammatory arthritis. Several kinds of pharmacological agents that modulate TLRs and downstream proteins have been shown to have in vitro, in vivo or clinical therapeutic effect in different inflammatory arthritis pathologies. Ab: antibodies; SM: small-molecules; PEP: peptides; ON: oligonucleotides; miRNA: microRNA.

The innate immune system is the host’s first line of defense against assaulting pathogens. Several innate immune receptors (pathogen recognition receptors—PRRs-) recognize a broad repertoire of molecular constituents in pathogens (pathogen associated molecular patterns—PAMPs-) such as ssRNA, CpG DNA or lipoproteins. The family of PRRs characterized in more depth is the family of Toll-like receptors (TLRs). In humans, 10 TLRs have been described. They are located at the plasma membrane (TLR1, 2, 4, 5, 6) or in the endosomes (TLR3, 7, 8, 9; Figure 1). Some TLRs homodimerize, and others form heterodimers (e.g., TLR2 with TLR1 or TLR6). TLR10 is not expressed in mice, and in humans, it seems to have an inhibitory role in the activity of TLR2 in immune cells [1]. TLRs may recognize, in addition to PAMPs, host-ligands derived from cellular or tissue damage, the so-called damage-associated molecular patterns (DAMPS or alarmins). Some examples include serum amyloid A—SAA-, high mobility group box 1 -HMGB-1-, endogenous nucleic acids, citrullinated fibrinogen or hyaluronan fragments. In some cases, DAMPs may be ligated by several TLRs (also in combination) as is the case of hyaluronan [2] and HMGB-1 [3] which may be recognized by TLR2, TLR4 and TLR5. In this way, the same DAMP may prompt different types of innate immune responses in an injury and cell-type specific manner. This response is also steered by the properties and chemical modifications of the DAMP (e.g., acetylation and molecular weight variations of hyaluronan [4] or post-translational modifications of HMGB-1).

TLR activation leads to the interaction with adaptor proteins (i.e., MyD88—Myeloid differentiation primary response 88- and Mal/TIRAP—Toll-interleukin 1 receptor (IL-1) domain containing adaptor protein; or TRIF—TIR-domain containing adapter inducing interferon-ß- and TRAM—TRIF-related adaptor molecule-) via TIR (Toll-IL-1 receptor)-domain interactions. Next, the adaptor proteins recruit downstream proteins in the signalling cascade. For instance, MyD88 forms the Myddosome complex which is composed of IRAK4 (interleukin-1 receptor-associated kinase 4), IRAK1, IRAK2 and TRAF6 (TNF-receptor associated factor 6) [5]. The IRAK1/TRAF6 complex dissociates from the receptor and interacts with TAK1 (TGF-β activated kinase 1) and TAB1/TAB2 (TAK1-binding proteins), which in turn bind E3 ligases Ubc13 and Uev1A [6]. Once the kinase TAK1 has been activated, it phosphorylates the IκB-kinase (IKK) complex (IKK-α, IKK-β and IKK-γ) and the mitogen activated protein kinases (MAPKs: extracellular signal-regulated kinase (ERK) 1/2, C-Jun N-terminal kinase (JNK) and p38). The final activation step in the signalling cascade leads to the translocation into the nucleus of several transcription factors (i.e.,: nuclear factor kappa B—NF-kB-, interferon regulatory factor—IRF- and activator protein 1—AP1-) which prompt the pro-inflammatory cytokine response (e.g., TNFα, IL-1ß) [7]. Additionally, NF-kB mediates the expression of cyclooxygenase 2 (COX-2) in several kinds of cells (e.g., rheumatoid synovial fibroblasts) [8]. COX-2 is one of the enzymes that regulates the production of the pro-inflammatory prostaglandin PGE2 from arachidonic acid (AA) released from the membrane by phospholipase A_2_ (PLA_2_). AA is also metabolized by lipoxygenases (e.g., 5-LOX) to produce the potent chemotactic agents leukotrienes (LT). It has also been shown that COX-2 expression can be induced by pro-inflammatory cytokines and mitogens (e.g., TNF-α, IL-1β, INF-γ) [9] and that certain PAMPs lead to the production of leukotrienes and prostaglandins in a cell specific manner [10].

In normal conditions, after instigation of the inflammatory response, a regulatory program to regain homeostasis is initiated (e.g., clearance of cellular debris, tissue repair), however, in inflammatory arthritis this activity is disrupted leading to a sustained inflammatory loop responsible for the chronic progression. In order to modulate inflammation, TLRs and their downstream proteins are explored as potential therapeutic targets. The next sections recapitulate the role of TLRs in various inflammatory pathologies, and also the kinds of compounds (e.g., small molecules, antibodies, peptides, oligonucleotides, micro RNAs) that have been discovered and engineered to control TLR expression and activity.

## 2. Rheumatoid Arthritis (RA)

RA is an autoimmune disease characterized by painful synovial inflammation, joint destruction, systemic inflammation and generation of autoantibodies (e.g., rheumatoid factor and anti-citrullinated protein antibodies) which affects 1% of the population worldwide [11]. Cartilage and bone damage in RA are associated with elevated TNFα levels produced by T-, B-cells, synovial-like fibroblasts and macrophage-like synoviocytes. Subsequently, other pro-inflammatory cytokines such as IL-6 and IL-1 are produced.

The aetiology of RA is still not completely understood. Lymphocytes and macrophages attack the synovium that in turn thickens and, if left untreated, invades and destroys the articular cartilage and underlying bone via proteolytic cleavage of aggrecan and collagen. Further, synovial fibroblasts secrete pro-inflammatory cytokines and matrix metal proteases (MMPs) what initiates the process of chronic inflammation. Several studies have emphasized the role of TLR activation in the abnormal synovial cell behavior [11]. It has been shown that the activation of TLR2 in synoviocytes leads to PLA_2_ activation, AA release and PGE2 production [12]. In turn, PGE2 mediates the increase in MMP and IL-1 expression amplifying the local inflammatory process. Likewise, LTs are important mediators of chronic inflammation and joint destruction in experimental models of RA, and leukotriene B4 (LTB4) levels are elevated in patients with RA [13,14].

The main therapy for RA patients consists of disease-modifying antirheumatic drugs (DMARDs), primarily methotrexate alone or in combination with glucocorticoids. Biological drugs that suppress the action of inflammatory cytokines (e.g., IL-1ß and TNFα) are used when DMARDs are not effective or toxic, however some patients do not respond well to these therapies.

While it is recognized that the major environmental factor that increases the risk for RA and the severity of the disease is smoking [15], 50% of the risk for RA development is due to genetic factors [11]. Thus, polymorphisms in TLR2, TLR4, TLR9 and NF-kB genes were associated with disease susceptibility or predisposition and response to TNFα treatment [16]. In contrast, allelic variants in TLR1, TLR7 and TLR8 were not found to be associated with susceptibility to RA. However, the variant M1V in TLR8 was associated with a lower need for DMARDs and biologic treatments due probably, to reduced production of inflammatory cytokines following TLR8 stimulation, indicating a protective role of this mutation [17]. Further, a TLR10 variant (I473T) led to increased NF-kB activity and was associated with disease severity and low response to infliximab (anti-TNFα antibody) [18].

The expression of different TLRs in cell-subsets implicated in RA development have been widely studied and TLR gene expression profiles are strongly associated with disease status. Clanchy et al. demonstrated that increased TLR expression in blood cells precedes clinical manifestation [19]. One study reported increased expression of TLR10 in B cell subsets and the levels correlated with the patients´ disease severity [20]. TLR1, 4 and 6 expression was reduced in whole blood after TNFα blocking and TLR2 expression in monocytes of patients who were non-responders to anti-TNFα therapy was higher compared to responders. Further, TLR3 and TLR7 expression was higher in synovial tissue of RA patients, indicating a possible involvement of viruses in the pathogenesis of RA [21] and several studies pointed to a pathological role of TLR9 and endogenous DNA in the disease [22]. As a consequence, inhibition, downregulation or neutralization of TLR function might have a positive therapeutic effect in RA as demonstrated in experimentally induced arthritis in mice. TLR4 and TLR5 neutralization with antibodies in that model reduced disease severity [23,24]. Furthermore, cytokine and MMP production by synovial tissue and synovial membrane cells from RA patients was inhibited by TLR2, TLR4 and TLR8 antibodies [25,26]. Likewise, TLR9 appears to be a possible target in RA as the TLR9 antagonist hydroxychloroquine halts the progression of RA by inhibiting dendritic cell (DC) maturation and migration from peripheral blood to the lymph nodes. Healthy DCs stimulated with serum from RA patients were treated with hydroxychloroquine, what led to lower expression of activation markers (i.e., CD86, CXCR4) and lower IFN-α secretion due to downregulation of TLR9 [27].

In contrast to the former reports, Monnet et al. demonstrated in a placebo-controlled, double-blind, randomized study, with non-responders to methotrexate, that blockage of TLR4 with NI-0101 (monoclonal anti-TLR4) did not produce a significant response in clinical endpoints or changes in inflammatory cytokines levels, in comparison with placebo effect [28]. The mechanism of action of methotrexate seems to be due to the upregulation of A20 expression (a negative regulator of NF-kB after TLR or TNFα-R stimulation) what impairs macrophage inflammatory responses [29]. Likewise, lower expression of the TLR negative regulator SARM (sterile-a and armadillo motif-containing protein) was found to correlate with higher disease activity and responders to anti-TNFα therapy showed upregulation of SARM in contrast to non-responders [30].

Several DAMPs have been found in the synovial membrane and fluid of RA patients. For instance, extracellular HMGB-1 is a DAMP that has been shown to activate TLR2, TLR4 [31] and TLR5 [32]. HMGB-1 expression in synovial tissue, fluid and serum of RA patients is elevated [33] pointing to the blockade of the cognate receptors as potential targets [34].

## 3. Spondyloarthropathies

Spondyloarthridities (SpA) are a group of inflammatory rheumatic diseases with complex aetiology that comprises psoriatic arthritis (PsA), seronegative reactive arthritis (ReA), arthritis associated with inflammatory bowel disease (IBD) and the most severe subtype, ankylosing spondylitis (AS). Patients present with inflammation of the spine and peripheral joints, and in some cases, extra-articular inflammatory manifestations such as psoriasis, uveitis or inflammatory bowel disease [35]. Abnormal host response against bacteria has been implicated in the pathogenesis of SpA, a disease that is characterized by abundant synovial infiltration of innate immune cells. It has been proposed that in genetically susceptible individuals, a first TLR stimulation by bacterial components, followed by sensitization to endogenous antigens that mimic bacterial products might lead to a persistent and chronic activation of the innate and adaptive immunity [36].

SpA has a strong genetic link with the human leukocyte antigen (HLA)-B27 marker [37]. Additionally, polymorphisms in TLR genes that lead to changes in these receptors or that affect the transcription of TLRs mRNA may be involved in the exaggerated inflammatory response observed in SpA [38]. Although a study with a reduced number of patients suggested that C-reactive protein and TLR4 gene polymorphisms may be related to the development of psoriatic arthritis, two larger studies with Brasilian and Korean populations showed no association between TLR4 polymorphisms and AS susceptibility [39,40,41]. Recently, a study with a broad patient cohort by Oliveira-Toré et al. demonstrated that certain polymorphisms in TLR2 and TLR9 increased 10 and 1.69-fold, respectively, the susceptibility to develop SpA, independently of the presence of antigen HLA-B27 [38]. This study suggests that the two receptors may contribute to the immunopathogenesis of SpA, and they may be potential therapeutic targets in spondyloarthritis. Additional hints to a possible implication of TLRs in SpA development comes from studies that show upregulated TLR2 expression in antigen presenting cells (monocytes and dendritic cells) of patients with PsA [42,43], and of TLR4 and TLR5 in AS patients [44]. Moreover, it has been shown that TNFα blockade down-modulates the increased systemic and local expression of TLR2 and TLR4 in SpA [45]. The causal relation for this elevated expression is not clear and it has been proposed that TLR up-regulation might be a non-specific indicator of activation of a more general inflammatory response in SpA [36].

## 4. Systemic Lupus Erythematosus (SLE)

SLE is an autoimmune disease characterized by the loss of tolerance to self-nuclear antigens, resulting in chronic systemic inflammation of the joints and various organs such as the kidneys and the brain. The organism produces antinuclear autoantibodies in a combined adaptive and innate immune response to endogenous nucleic acids released after cell death. Defective clearance mechanisms (e.g., enhanced apoptosis and neutrophil extracellular traps, nucleic acid debris) trigger the activation of the innate immune system and the production of interferons [46]. The current therapy comprises NSADs (non-steroidal anti-inflammatory drugs), hydroxychloroquine, immunosuppressants and short courses of corticosteroids.

Numerous studies have exposed the predominant role of endosomal TLRs in the detection of host DNA and RNA in SLE [47], but also TLR2 and TLR4 seem to be upregulated in peripheral blood mononuclear cells of SLE patients [48].

However, no association has been found between SLE and common polymorphisms in TLR2 (R677W and R753Q) and TLR4 (D299G and T399I) [49]. In contrast, the association between clinical manifestations and TLR7, TLR8 and TLR9 polymorphisms is clear [50,51] and the involvement of the endosomal TLRs, mainly TLR7, in lupus is backed up by several studies [52]. The over-expression of TLR7 in SLE patients is detrimental and conversely, some reports suggest that TLR8 and TLR9 have a protective role in the TLR7 response to RNA-associated autoantibodies in dendritic cells [53,54]. Various oligonucleotides that mimic TLR ligands (for TLR7, TLR8, TLR9) have been synthesized and are prospective therapeutics for lupus [55].

## 5. Systemic Juvenile Idiopathic Arthritis and Adult-Onset Still’s Disease

Systemic juvenile idiopathic arthritis (sJIA), which affects children between 1 and 5 years of age, and adult-onset Still’s disease (AOSD) are systemic inflammatory disorders characterized by spiking fever, joint pain, skin rash, hepato- and/or spleno-megaly and leukocytosis. Even though the pathophysiology remains unknown, several hints point to a dysregulated immune system: elevated pro-inflammatory cytokine levels and acute phase markers, rapid clinical response to IL-1-blocking strategies and the absence of autoantibodies [56]. To this respect, Chen et al. [57] observed that the levels of TLR7 were elevated and positively correlated with disease activity after evaluating TLR7 expression in DCs from AOSD patients. It seems clear that polymorphisms in single genes do not cause sJIA, however, most confirmed genetic associations involve pro- or anti-inflammatory cytokine genes [56]. In addition, environmental factors and several DAMPs seem to play a role in the development or severity of systemic JIA and AOSD [58]. The concentration of S100 proteins (S100A8/A9) correlates with response to treatment and disease activity in SJIA patients [59], and HMGB1 [60] and SAA [61] serum levels are elevated in JIA and AOSD patients, and downregulated after disease resolution. Dysregulated release of DAMPS might lead to TLR activation (e.g., TLR4 in the case of S100, HMGB1 and SAA) and initiation or maintenance of the inflammatory response. Accordingly, cytokine blocking strategies (e.g., anti-IL-1, anti-IL6) are implemented with sJIA patients usually after an initial high-dose of corticosteroid treatment or when the corticosteroid tapering fails [56].

## 6. Synthetic Ligands That Down-Regulate TLR Activity

In the last decade, different kinds of inhibitors targeting the TLR pathways have been designed. Those with a potential therapeutic use in inflammatory arthritis will be described in the next sections. Some of the inhibitors targeted the binding of the activating ligand to the receptor and others the intracellular signalling triggered after TLR ligation. The first kind of inhibitors are selective for a defined TLR pathway, while the last group of substances may inhibit several signalling cascades simultaneously. Intracellular inhibitors are either cell permeable per se or they must be modified in order to transverse the cell plasma membrane and reach their target (e.g., nanocarriers, linkage with cell-penetrating peptides). Intracellular targets might be proteins proximal to the receptor (e.g., adaptors such as MyD88, TIRAP) or further down in the signalling cascade (Figure 1). Most TLR pathways, except TLR3, share the adaptor MyD88. Other inflammatory pathways, such as the TNFα-R pathway, have only some proteins in common with the TLR-pathway (e.g., TAK1/TAB1).

Thus, inhibitors that target downstream proteins display broader anti-inflammatory properties since they downregulate several signalling cascades simultaneously. On the other hand, down regulation of a large part of the inflammatory response might leave the body unguarded against the outbreak of pathogen infections.

### 6.1. TLR Blocking Antibodies

Therapeutic antibodies are highly specific drugs with lower off-target effects and have a longer half-life in comparison with other drugs. However, their production is costly, they have low cellular and tissue penetration, and they are potentially immunogenic [62]. Since the early days in the TLR history, when antibodies were used to block in vitro ligand recognition (e.g., TL2.1, T2.5, 1A6) [63,64], several therapeutic antibodies have been developed (Table 1). OPN-305 is the first fully humanized IgG4 monoclonal TLR2 specific antibody, which has shown promising results in clinical studies, until now, in organ transplantation [65]. NI-0101 is the first monoclonal anti-TLR4 antibody used in clinical trials. It hinders TLR4 dimerization and it blocks pro-inflammatory cytokine production in monocytes stimulated with synovial fluid from RA patients. NI-0101 has been tested in clinical trials in RA patients [28], unfortunately without showing any benefit.

**Table 1:** TLR inhibitors used in inflammatory arthritis.

Disease	Target	Pharmacological Agent	
		In Vitro/In Vivo	Clinical Trial (for This Indication)
**RA**	TLR2	ntibody: OPN301 [26] miRNA: miR-149a/b [66]	
	TLR3	miRNA: miR-26a-5p [67]	
	TLR4	Peptide: PIP2 [68]	
		Small molecule: TAK-242 [69]	
			Antibody: NI-0101 [28] Non effective
	TLR5	Antibody [24]	
	TLR8	Small molecule: Mianserin, chloroquine, imiquimod [25]	
	TLR9	Small molecule: Hydroxychloroquine [27]	
	TLR7/8	MicroRNA: miR574-5p [70,71,72]	
	TLR7/8/9	Small molecule: IMO-9200 [73]	
	IRAK4		Small molecule: PF-0665033 [74]
	p38	Small molecule: Org48762-0 [75]	
	IKKβ	Small molecule: CHPD [76]	
	A20 (negative regulator) [29]	-	
	SARM (negative regulator) [30]	-	
	HMGB-1 (TLR2-, TLR4-, TLR5-ligand)	Antibodies [34]	
**SpA**	TLR2? [38]		
	TLR4? [45]		
	TLR5? [44]		
	TLR9? [38]		
**SLE**	TLR9		Small molecule: Hydroxychloroquine [77] Effective
	TLR7/9	Oligonucleotides: IRS-954 [78]	
			Oligonucleotides: DV-1179 [79] Non effective
	TLR7/8/9	Oligonucleotides: IMO-8400 [80]Small molecule: Compound f [81]	
			Small molecule: CpG-52364 [82]
	IRAK1/TRAF6	MicroRNA: miR-146a [83]	
**JIA/AOSD**	TLR7 high expression [57]		
	S100 elevated in serum [84]/TLR4?		
	HMGB-1 elevated in serum [60,85]/TLR4?		
	SAA elevated in serum [61]/TLR4?		

It seems more challenging to develop antibodies with inhibitory function for endosomal TLRs. Nonetheless, Fukui and collaborators [86] were able to obtain antibodies against a complete panel of TLRs (TLR1-9), some of them with blocking function (e.g., TLR3: TLR3.7; TLR4/MD-2: MTS510, Sa15-21; TLR7: A94B10; TLR9: NaR9). The authors reasoned that the mechanism by which the antibodies blocked endosomal TLRs is that some of these are also expressed in the plasma membrane, because antibody uptake is Fc receptor-dependent and the TLR7 antibody A94B10 could be internalized also in the absence of Fc receptor. A94B10 reduces the systemic inflammation caused by TLR7 hyper-response and NaR9 inhibited TLR9-dependent lethal hepatitis in mice, thus, these antibodies appear as a promising alternative treatment in SLE, RA or psoriasis [87].

### 6.2. Oligonucleotides

Endosomal TLR receptors recognize ssRNA, dsDNA and CpG-DNA, thus oligonucleotide inhibitors that mimic the original ligand and bind to the receptor, block TLR signalling. In this way, tri-functional TLR7/8/9 (IMO-8400) [80], bi-functional TLR7/9 (IRS-954; DV-1179; IMO-3100; INH-ODN-24888) or selective TLR7 (IRS-661) [88] and TLR9 (IRS-869) [89] inhibitors have been developed. IMO-8400 (Immune Modulatory Oligonucleotide-8400; Idera Phamaceuticals) inhibited NF-kB activation and production of pro-inflammatory cytokines in a mouse model of SLE and showed therapeutic effect in patients with moderate to severe plaque psoriasis [90]. IRS-954 (Immunoregulatory DNA sequence-954; Dynavax Technologies) inhibited the induction of IFN-α by human pDCs in response to DNA and RNA viruses and isolated immune complexes from lupus patients [91]. In in vivo studies, IRS-954 reduced the serum levels of nucleic acid-specific autoantibodies, proteinuria, glomerulonephritis, end-organ damage and increased survival [78]. DV-1179 (Dynavax Technologies) has been tested in clinical studies, however, it did not satisfy the pharmacodynamic endpoints related to reduction in IFN-α-regulated genes [79]. INH-ODN-24888 (Inhibitory Oligonucleotide-24888) reduces TLR7/9 mediated immune responses in human immune cells and is a promising therapeutic agent for the treatment of SLE [92].

### 6.3. Peptides

Targeting protein-protein interactions with small molecules is challenging due to the size and relatively flat and featureless topologies of the interacting surfaces involved [93]. One strategy is to design decoy peptides that resemble the interaction surface of one protein with its partner. With the purpose of inhibiting TLR2 signalling, Ebner et al. [94] designed a collection of peptides derived from the extracellular domain of TLR2. The overlapping peptides covered epitopes involved either in TLR2/TLR1 hetero-dimerization, or in interaction with the tri-acylated lipopeptide ligand Pam3CySK4 (among leucine-rich repeats—LRR- 11 and 13), as indicated by structural studies. The decoy peptides decreased selectively the TLR2/TLR1 mediated inflammatory response in human and mouse cells. In addition, by means of phage display, Achek et al. [68] selected a peptide (PIP2) that inhibited TLR4 signalling by interfering with the TLR4/MD2 interaction, and also with the activity of other TLRs (TLR1/2/6 and TLR7/8/9) although with lower affinity. PIP2 relieved RA symptoms and displayed a protective effect in an RA rat model.

In order to target intracellular protein-protein interactions, the peptides have to pass through the plasma membrane, for example, when they are linked to a cell penetrating peptide (e.g., penetratin, TAT -transactivating transcriptional activator peptide–, antennapedia). Yet, this method of molecular delivery is unspecific. The first peptide derived from the BB-loop of the TIR domain of the adaptor TIRAP was fused to penetratin and it inhibited LPS-induced NF-kB activation in mouse macrophages [95]. Likewise, a peptide derived from the BB loop of MyD88 (ST2345) interfered with the dimerization of MyD88. The synthetic peptide was connected to Antennapedia, and it showed inhibitory activity on IL-1 mediated NF-kB activity [96].

Furthermore, peptides with inhibitory function derived from microbial proteins have been used. Microbial pathogens use proteins that can interact with TLR-signalling proteins to avoid the TLR-mediated immune response [97]. A peptide derived from the protein A52R from vaccinia virus reduced the cytokine production following TLR3, TLR4 or TLR9 stimulation in RAW264.7 macrophages [98]. Until now, this kind of peptides has been mainly used in vitro or in mouse models of infection and sepsis [99].

### 6.4. Small Molecules

Small molecules present some advantages in comparison to the above-described inhibitors. They can be taken orally, and depending on their chemico-physical properties, they can penetrate the cell membrane to target intracellular proteins, besides, their manufacturing is cheaper. The TLR7/8/9 small molecule antagonists hydroxychloroquine sulfate, chloroquine and quinacrine were initially used as antimalarial drugs, and later on, applied in the treatment of SLE [77]. Afterwards, triple (TLR7/8/9) [81], double (TLR7/8 [100], TLR7/9 [101,102]) and selective (TLR8 [103] or TLR7 [104]) antagonists have been synthesized. The tri-functional inhibitor compound *f* (a novel orally available 2-phenyl indole derivative) showed anti-inflammatory and good pharmacokinetic properties in preclinical models of lupus and psoriasis [81]. CpG-52364 (Coley Pharmaceutical) is also a TLR7/8/9 inhibitor, which works better than hydroxychloroquine sulfate in animal studies and it was tested in clinical trials for the oral treatment of SLE, although no results to the clinical study (NCT00547014; 2009) were posted [82]. IMO-9200 (TLR7/8/9) showed promising in vivo results in inflammatory bowel disease, and it entered phase I clinical trials with a good safety profile. However, the drug was outsourced in 2016 from Idera Pharmaceuticals to Vivelix Pharmaceuticals and no subsequent data have been reported [73].

Our group [105] has discovered several small molecule inhibitors of TLR2/TLR1 and TLR2/TLR6 by computer-aided drug design and in vitro screening. One of those compounds, AT5, decreased the TNFα and IL-6 production in a mouse model of lipopeptide-induced inflammation [106]. Further small molecules targeting TLR2/TLR1 have been reported (CU-CPT22 [107], SMU-Z1 [108]). The TLR4 inhibitor TAK-242 (Resatorvid) has been tested in in vitro and in vivo models of RA. It has attained successful decrease of LPS-mediated expression of IL-6, IL-8, MMP-1 and VEGF and it ameliorates the inflammatory symptoms of joint tissues in a rat model of arthritis [69]. In contrast, another selective TLR4 antagonist, T5342126, showed strong non-specific effects (e.g., decreased animal locomotor activity) in a mice model of ethanol dependence what precludes its further use [109].

Several groups have reported inhibitors that target the proteins downstream in the TLR signalling cascades. IRAK4- or MyD88-deficient patients suffer from bacterial or viral infections but not from autoimmune diseases, suggesting that targeting of IRAK4 and MyD88 may prevent autoimmunity in humans [110]. Thus, the dimerization of MyD88 or its interaction with downstream proteins have been targeted via peptidomimetics (e.g., ST-2825) [111]. Moreover, various IRAK1/4 kinase inhibitors have been developed [112], among them, an N-acyl2-aminobenzimidazole derivative abrogated the TLR7/9-induced IFN-α responses in both, mouse and human pDCs [113]. Others (PF-05387252, PF-05388169, AS-2444697 and PF-0665033) are tested in preclinical- and phase I clinical-studies with favorable safety and pharmacokinetic profiles. PF-0665033 was the first IRAK4 inhibitor to enter clinical development, and it is tested in clinical trials for rheumatic and autoimmune diseases [74].

More downstream, it has been shown that MAPKs (p38 and JNK) and the kinases that regulate them (TAK1, MEKK-2, MKK-4, MKK-7) are activated in macrophages and fibroblasts of the synovial lining layer and at sites of bone erosion [114]. Inhibition of p38 seems effective in suppressing joint destruction and TNF-α release in RA disease models [75]. However, in spite of the development of selective inhibitors, problems of toxicity in liver, skin and/or central nervous system have been reported, probably due to the fact that p38 plays a central role in muscle differentiation, erythropoiesis and bone formation. Overall, the feasibility of a therapeutic use of p38 inhibitors is under dispute [115].

IKKs (inhibitory kappa B kinases) have been a target of interest since long, and several groups and pharmaceutical companies have developed very potent and relatively selective IKKα and IKKβ inhibitors (e.g., BMS-345541, Bristol-Myers Squibb [116]). Yet, also in this case, several problems of toxicity have been reported [117]. Drexel at al [118] described the discovery of INH14, a phenylurea derivative that inhibits IKKα/β. In vitro experiments indicated that INH14 decreased TLR2-, TLR4-, TNFα-R- and IL-1R-mediated inflammatory activity. In a mouse model of lipopeptide-induced inflammation, INH14 treatment led to a decrease in TNFα production. The authors did not observe toxicity at the concentrations tested. Further, Tsuchiya et al. [76] reported that CHPD, a selective IKKβ inhibitor, strongly reduced the production of inflammatory cytokines (IL-6 and IL-8) in rheumatoid synovial fibroblasts.

Classical non-steroidal anti-inflammatory drugs (NSAIDs; e.g., diclofenac) are inhibitors of COX-2 and they are applied in RA to decrease inflammation and alleviate pain, yet they inhibit also COX-1 and they can produce gastrointestinal toxicity. Accordingly, great effort has been invested in the selection of selective COX-2 inhibitors (Coxibs: celecoxib, rofexocib, etericoxib). Disappointingly, these drugs presented an increased cardiovascular risk. Lately, in order to reduce the production of leukotrienes in addition to prostaglandins, dual inhibitors of COX-2/5-LOX have been developed (e.g., Tenidap—Pfizer-) however, they showed an unfavorable toxicity profile and studies to identify new pharmacophore models are on the way [119].

Finally, nanoformulations of small molecules in gold or silver nanoparticles present some advantages such as passive or active delivery of the drugs to the target cells or subcellular domains, besides improving their aqueous solubility and the protection from enzymatic degradation. Nanoparticles of the α-pyrones opuntiol and opuntioside showed anti-arthritic activity downregulating IL-1β, TNFα, TLR2 and TLR4 expression [120].

### 6.5. Micro RNAs

Lately, the study of the regulation of TLRs by microRNAs (miRNAs) has been a flourishing field [121]. miRNAs are small (21-25 nucleotides), non-coding, regulatory RNAs which bind to a sequence within the 3′ untranslated region (UTR) of the mRNA from target proteins, promoting the degradation or the inhibition of translation of the mRNA [122]. Several miRNAs that regulate TLR pathways have been identified and are potential targets to treat inflammatory diseases, and different companies are developing agomirs or antagomirs (modified microRNAs, for instance with cholesterol, to improve their chemical properties) targeting mRNAs. However, miRNAs can regulate more than one protein, thus unwanted effects might be attained by using them as therapeutic agents.

In fibroblast-like synoviocytes the elevated expression of TLR2 can be regulated by miR-19a/b, what leads to decreased IL-6 production [66] and a decrease of TLR3 mRNA expression by miR-26a-5p in arthritic rats slowed the development of RA [67]. Micro RNAs for other TLRs have been also developed: TLR4 (miR-100-5p), TLR6 (miR-124-5p), TLR7 (miR-150-5p, miR-152-5p, miR-375-5p). In addition, several miRNAs regulate the expression of MyD88 (MiR-155-5p [123], miR-203-5p [124], miR-149-5p [125], miR-124-5p [126]) and their overexpression leads to inhibition of IL-6 and TNFα. IRAK1 is targeted by various miRNAs (miR-21-5p, miR-133-5p, miR-142-3p, miR146a/b-5p). MiR-146a downregulates the production of type I IFNs in human lupus by targeting simultaneously TRAF6 and IRAK1 [83]. In SLE and RA patients, a reduced expression of miR-23b-5p and accordingly higher expression of TAB2, TAB3 and IKKα has been observed, and in turn, increased production of the cytokines TNFα, IL-1β and IL-17 [127].

Abnormal expression of micro RNAs (miRNAs) play a prominent role in the maintenance of RA [128]. It has been shown that enhanced osteoclast maturation is mediated by TLR7/8 signalling when they are activated by miR-574-5p. This is a non-coding RNA carried by cell-derived small extracellular vesicles that mediate cell-to-cell communication in the synovial microenvironment. This novel mechanism underlying the pathogenesis of RA points to miR-574-5p as a target to protect against osteoclast mediated cartilage destruction [70,71]. Further, a MiR-147 mimic suppressed the expression of TLR7 in a pristane-induced arthritic rat model and improved the severity of arthritis [72]. These reports evidence that microRNAs can directly regulate activation of TLRs and might be important drug-targets [129].

## 7. Conclusions

The involvement of plasma membrane located TLRs in RA, and of endosomal TLRs in SLE progression has been broadly reported. However, whether TLRs play any role in the pathology of SpA is, at least, uncertain. Concrete hints indicate that host-derived DAMPs are elevated in IA/AOSD, but whether this is the cause or the consequence of the underlying inflammatory process needs to be elucidated.

It seems reasonable to conclude that antagonists modulating TLR activity at the level of the receptor might show lower anti-inflammatory potential as inhibitors targeting downstream proteins implicated in several pathways (e.g., TLRs, IL-1R, TNFα-R). The last inhibitors might, at least theoretically, lead to a more widespread, and perhaps potent, systemic inhibition of cytokine production. However, experimental evidence shows that the more downstream the inhibitory intervention points to, the greater the toxicity issues (e.g., p38 [67]). In addition, inhibition of several inflammatory pathways might leave the host partially unprotected in the likely case of a bacterial or viral infection. This leads irredeemably to enquiry whether it is safe to use more than one TLR inhibitor (or one inhibitor for different TLRs) instead of pointing to a single target. At least, a successful example of multi-targeting endosomal TLRs is evidenced by the in vivo results in SLE and RA models (Table 1). By extension, as various host-derived DAMPs are involved in the pathologies of RA or IA/AOSD, it is tempting to speculate, that inhibition of several TLRs (in general targets) might prove to be a more effective anti-inflammatory strategy. Certainly, it is to be kept in mind that off-target effects and synergisms may obscure the therapeutic outcome.

No specific TLR inhibitors have yet been approved for any of the described indications, except the antimalarial compounds that target endosomal TLRs and that are used to treat RA and SLE [130]. From the developmental point of view, small molecule TLR inhibitors would be advantageous for the treatment of inflammatory arthritis in comparison to biologics, as their production is more economical and anti-drug antibodies will not develop in the treated patients, yet, new promising ways of intervention are emerging, such as the regulation of/by micro RNAs.

In summary, targeting TLR pathways to effectively decrease the immune response in chronic inflammatory diseases needs a deeper understanding of the mechanisms and dynamics of TLR activation in the different pathologies, besides extensive investigation of the side effects that TLR modulation might cause in a sustained therapy.

## References

[1] Oosting M., Cheng S.C., Bolscher J.M., Vestering-Stenger R., Plantinga T.S., Verschueren I.C., Arts P., Garritsen A., van Eenennaam H., Sturm P. (2014). Human TLR10 is an anti-inflammatory pattern-recognition receptor. Proc. Natl. Acad. Sci. USA.

[2] Hussain S., Johnson C.G., Sciurba J., Meng X., Stober V.P., Liu C., Cyphert-Daly J.M., Bulek K., Qian W., Solis A. (2020). TLR5 participates in the TLR4 receptor complex and promotes MyD88-dependent signaling in environmental lung injury. Elife.

[3] Park J.S., Gamboni-Robertson F., He Q., Svetkauskaite D., Kim J.Y., Strassheim D., Sohn J.W., Yamada S., Maruyama I., Banerjee A. (2006). High mobility group box 1 protein interacts with multiple Toll-like receptors. Am. J. Physiol. Cell Physiol..

[4] Tighe R.M., Garantziotis S. (2019). Hyaluronan interactions with innate immunity in lung biology. Matrix Biol..

[5] Lin S.C., Lo Y.C., Wu H. (2010). Helical assembly in the MyD88-IRAK4-IRAK2 complex in TLR/IL-1R signalling. Nature.

[6] Wang C., Deng L., Hong M., Akkaraju G.R., Inoue J., Chen Z.J. (2001). TAK1 is a ubiquitin-dependent kinase of MKK and IKK. Nature.

[7] Takeda K., Akira S. (2004). TLR signaling pathways. Semin. Immunol..

[8] Crofford L.J., Tan B., McCarthy C.J., Hla T. (1997). Involvement of nuclear factor kappa B in the regulation of cyclooxygenase-2 expression by interleukin-1 in rheumatoid synoviocytes. Arthritis Rheum..

[9] Arias-Negrete S., Keller K., Chadee K. (1995). Proinflammatory cytokines regulate cyclooxygenase-2 mRNA expression in human macrophages. Biochem. Biophys. Res. Commun..

[10] Alvarez Y., Valera I., Municio C., Hugo E., Padrón F., Blanco L., Rodríguez M., Fernández M.N., Crespo M.S. (2010). Eicosanoids in the Innate Immune Response: TLR and Non-TLR Routes. Mediat. Inflamm..

[11] Scott D.L., Wolfe F., Huizinga T.W. (2010). Rheumatoid arthritis. Lancet.

[12] Sommerfelt R.M., Feuerherm A.J., Skuland T., Johansen B. (2015). Cytosolic Phospholipase A2 Modulates TLR2 Signaling in Synoviocytes. PLoS ONE.

[13] Martel-Pelletier J., Pelletier J.P., Fahmi H. (2003). Cyclooxygenase-2 and prostaglandins in articular tissues. Semin. Arthritis Rheum..

[14] Bertolini A., Ottani A., Sandrini M. (2001). Dual acting anti-inflammatory drugs: A reappraisal. Pharmacol. Res..

[15] Elshabrawy H.A., Essani A.E., Szekanecz Z., Fox D.A., Shahrara S. (2017). TLRs, future potential therapeutic targets for RA. Autoimmun. Rev..

[16] Gebura K., Swierkot J., Wysoczanska B., Korman L., Nowak B., Wiland P., Bogunia-Kubik K. (2017). Polymorphisms within genes involved in regulation of the NF-kappaB pathway in patients with rheumatoid arthritis. Int. J. Mol. Sci..

[17] Torices S., Alvarez-Rodriguez L., Varela I., Munoz P., Balsa A., Lopez-Hoyos M., Martinez-Taboada V., Fernandez-Luna J.L. (2017). Evaluation of Toll-like-receptor gene family variants as prognostic biomarkers in rheumatoid arthritis. Immunol. Lett..

[18] Torices S., Julia A., Munoz P., Varela I., Balsa A., Marsal S., Fernandez-Nebro A., Blanco F., Lopez-Hoyos M., Martinez-Taboada V. (2016). A functional variant of TLR10 modifies the activity of NFkB and may help predict a worse prognosis in patients with rheumatoid arthritis. Arthritis Res. Ther..

[19] Clanchy F.I.L., Borghese F., Bystrom J., Balog A., Penn H., Hull D.N., Wells G.M.A., Kiriakidis S., Taylor P.C., Sacre S.M. (2021). TLR expression profiles are a function of disease status in rheumatoid arthritis and experimental arthritis. J. Autoimmun..

[20] Zhang Y., Cao R., Ying H., Du J., Chen S., Wang N., Shen B. (2018). Increased expression of TLR10 in B cell subsets correlates with disease activity in rheumatoid arthritis. Mediat. Inflamm..

[21] Roelofs M.F., Joosten L.A., Abdollahi-Roodsaz S., van Lieshout A.W., Sprong T., van den Hoogen F.H., van den Berg W.B., Radstake T.R. (2005). The expression of Toll-like receptors 3 and 7 in rheumatoid arthritis synovium is increased and costimulation of Toll-like receptors 3, 4, and 7/8 results in synergistic cytokine production by dendritic cells. Arthritis Rheum..

[22] Fischer A., Abdollahi-Roodsaz S., Bohm C., Niederreiter B., Meyer B., Yau A.C.Y., Lonnblom E., Joosten L.A.B., Koenders M., Lehmann C.H.K. (2018). The involvement of Toll-like receptor 9 in the pathogenesis of erosive autoimmune arthritis. J. Cell Mol. Med..

[23] Abdollahi-Roodsaz S., Joosten L.A., Roelofs M.F., Radstake T.R., Matera G., Popa C., van der Meer J.W., Netea M.G., van den Berg W.B. (2007). Inhibition of Toll-like receptor 4 breaks the inflammatory loop in autoimmune destructive arthritis. Arthritis Rheum..

[24] Kim S.J., Chen Z., Chamberlain N.D., Essani A.B., Volin M.V., Amin M.A., Volkov S., Gravallese E.M., Arami S., Swedler W. (2014). Ligation of TLR5 promotes myeloid cell infiltration and differentiation into mature osteoclasts in rheumatoid arthritis and experimental arthritis. J. Immunol..

[25] Sacre S.M., Lo A., Gregory B., Simmonds R.E., Williams L., Feldmann M., Brennan F.M., Foxwell B.M. (2008). Inhibitors of TLR8 reduce TNF production from human rheumatoid synovial membrane cultures. J. Immunol..

[26] Ultaigh S.N., Saber T.P., McCormick J., Connolly M., Dellacasagrande J., Keogh B., McCormack W., Reilly M., O’Neill L.A., McGuirk P. (2011). Blockade of Toll-like receptor 2 prevents spontaneous cytokine release from rheumatoid arthritis ex vivo synovial explant cultures. Arthritis Res. Ther..

[27] Han J., Li X., Luo X., He J., Huang X., Zhou Q., Han Y., Jie H., Zhuang J., Li Y. (2020). The mechanisms of hydroxychloroquine in rheumatoid arthritis treatment: Inhibition of dendritic cell functions via Toll like receptor 9 signaling. Biomed. Pharmacother..

[28] Monnet E., Choy E.H., McInnes I., Kobakhidze T., de Graaf K., Jacqmin P., Lapeyre G., de Min C. (2002). Efficacy and safety of NI-0101, an anti-Toll-like receptor 4 monoclonal antibody, in patients with rheumatoid arthritis after inadequate response to methotrexate: A phase II study. Ann. Rheum. Dis..

[29] Municio C., Dominguez-Soto A., Fuentelsaz-Romero S., Lamana A., Montes N., Cuevas V.D., Campos R.G., Pablos J.L., Gonzalez-Alvaro I., Puig-Kroger A. (2018). Methotrexate limits inflammation through an A20-dependent cross-tolerance mechanism. Ann. Rheum. Dis..

[30] Thwaites R.S., Unterberger S., Chamberlain G., Gray H., Jordan K., Davies K.A., Harrison N.A., Sacre S. (2021). Expression of sterile-alpha and armadillo motif in rheumatoid arthritis monocytes correlates with TLR2 induced IL-1beta and disease activity. Rheumatology.

[31] Park J.S., Svetkauskaite D., He Q., Kim J.Y., Strassheim D., Ishizaka A., Abraham E. (2004). Involvement of Toll-like receptors 2 and 4 in cellular activation by high mobility group box 1 protein. J. Biol. Chem..

[32] Das N., Dewan V., Grace P.M., Gunn R.J., Tamura R., Tzarum N., Watkins L.R., Wilson I.A., Yin H. (2016). HMGB1 activates proinflammatory signaling via TLR5 leading to Allodynia. Cell Rep..

[33] Kaur I., Behl T., Bungau S., Kumar A., Mehta V., Setia D., Uddin M.S., Zengin G., Aleya L., Arora S. (2020). Exploring the therapeutic promise of targeting HMGB1 in rheumatoid arthritis. Life Sci..

[34] Kokkola R., Li J., Sundberg E., Aveberger A.C., Palmblad K., Yang H., Tracey K.J., Andersson U., Harris H.E. (2003). Successful treatment of collagen-induced arthritis in mice and rats by targeting extracellular high mobility group box chromosomal protein 1 activity. Arthritis Rheum..

[35] Diaz-Pena R., Castro-Santos P., Duran J., Santiago C., Lucia A. (2002). The genetics of spondyloarthritis. J. Pers. Med..

[36] Tan F.K., Farheen K. (2011). The potential importance of Toll-like receptors in ankylosing spondylitis. Int. J. Clin. Rheumtol..

[37] Schlosstein L., Terasaki P.I., Bluestone R., Pearson C.M. (1973). High association of an HL-A antigen, W27, with ankylosing spondylitis. N. Engl. J. Med..

[38] Oliveira-Tore C.F., Moraes A.G., Martinez G.F., Neves J.S.F., Macedo L.C., Rocha-Loures M.A., Quirino M.G., Alves H.V., Sell A.M., Visentainer J.E.L. (2019). Genetic polymorphisms of Toll-like receptors 2 and 9 as susceptibility factors for the development of ankylosing spondylitis and psoriatic arthritis. J. Immunol. Res..

[39] Akbal A., Oguz S., Gokmen F., Bilim S., Resorlu H., Silan F., Uludag A. (2015). C-reactive protein gene and Toll-like receptor 4 gene polymorphisms can relate to the development of psoriatic arthritis. Clin. Rheumatol..

[40] Machado N.P., Nogueira E., Oseki K., Ebbing P.C., Origassa C.S., Mohovic T., Camara N.O., Pinheiro M.M. (2016). Clinical characteristics and frequency of TLR4 polymorphisms in Brazilian patients with ankylosing spondylitis. Rev. Bras. Reumatol. Engl. Ed..

[41] Na K.S., Kim T.H., Rahman P., Peddle L., Choi C.B., Inman R.D. (2008). Analysis of single nucleotide polymorphisms in Toll-like receptor 4 shows no association with ankylosing spondylitis in a Korean population. Rheumatol. Int..

[42] Carrasco S., Neves F.S., Fonseca M.H., Goncalves C.R., Saad C.G., Sampaio-Barros P.D., Goldenstein-Schainberg C. (2011). Toll-like receptor (TLR) 2 is upregulated on peripheral blood monocytes of patients with psoriatic arthritis: A role for a gram-positive inflammatory trigger?. Clin. Exp. Rheumatol..

[43] Candia L., Marquez J., Hernandez C., Zea A.H., Espinoza L.R. (2007). Toll-like receptor-2 expression is upregulated in antigen-presenting cells from patients with psoriatic arthritis: A pathogenic role for innate immunity?. J. Rheumatol..

[44] Assassi S., Reveille J.D., Arnett F.C., Weisman M.H., Ward M.M., Agarwal S.K., Gourh P., Bhula J., Sharif R., Sampat K. (2011). Whole-blood gene expression profiling in ankylosing spondylitis shows upregulation of Toll-like receptor 4 and 5. J. Rheumatol..

[45] De Rycke L., Vandooren B., Kruithof E., De Keyser F., Veys E.M., Baeten D. (2005). Tumor necrosis factor alpha blockade treatment down-modulates the increased systemic and local expression of Toll-like receptor 2 and Toll-like receptor 4 in spondylarthropathy. Arthritis Rheum..

[46] Rose T., Dorner T. (2017). Drivers of the immunopathogenesis in systemic lupus erythematosus. Best Pract. Res. Clin. Rheumatol..

[47] Marshak-Rothstein A. (2006). Toll-like receptors in systemic autoimmune disease. Nat. Rev. Immunol..

[48] Lee Y.H., Choi S.J., Ji J.D., Song G.G. (2016). Association between Toll-like receptor polymorphisms and systemic lupus erythematosus: A meta-analysis update. Lupus.

[49] Sanchez E., Orozco G., Lopez-Nevot M.A., Jimenez-Alonso J., Martin J. (2004). Polymorphisms of Toll-like receptor 2 and 4 genes in rheumatoid arthritis and systemic lupus erythematosus. Tissue Antigens.

[50] Enevold C., Nielsen C.H., Jacobsen R.S., Hermansen M.L., Molbo D., Avlund K., Bendtzen K., Jacobsen S. (2014). Single nucleotide polymorphisms in genes encoding Toll-like receptors 7, 8 and 9 in Danish patients with systemic lupus erythematosus. Mol. Biol. Rep..

[51] Dos Santos B.P., Valverde J.V., Rohr P., Monticielo O.A., Brenol J.C., Xavier R.M., Chies J.A. (2012). TLR7/8/9 polymorphisms and their associations in systemic lupus erythematosus patients from southern Brazil. Lupus.

[52] Celhar T., Magalhaes R., Fairhurst A.M. (2012). TLR7 and TLR9 in SLE: When sensing self goes wrong. Immunol. Res..

[53] Nickerson K.M., Christensen S.R., Shupe J., Kashgarian M., Kim D., Elkon K., Shlomchik M.J. (2010). TLR9 regulates TLR7- and MyD88-dependent autoantibody production and disease in a murine model of lupus. J. Immunol..

[54] Desnues B., Macedo A.B., Roussel-Queval A., Bonnardel J., Henri S., Demaria O., Alexopoulou L. (2014). TLR8 on dendritic cells and TLR9 on B cells restrain TLR7-mediated spontaneous autoimmunity in C57BL/6 mice. Proc. Natl. Acad. Sci. USA.

[55] Kandimalla E.R., Bhagat L., Wang D., Yu D., Sullivan T., La Monica N., Agrawal S. (2013). Design, synthesis and biological evaluation of novel antagonist compounds of Toll-like receptors 7, 8 and 9. Nucleic Acids Res..

[56] Bruck N., Schnabel A., Hedrich C.M. (2015). Current understanding of the pathophysiology of systemic juvenile idiopathic arthritis (sJIA) and target-directed therapeutic approaches. Clin. Immunol..

[57] Chen D.Y., Lin C.C., Chen Y.M., Lan J.L., Hung W.T., Chen H.H., Lai K.L., Hsieh C.W. (2013). Involvement of TLR7 MyD88-dependent signaling pathway in the pathogenesis of adult-onset Still’s disease. Arthritis Res. Ther..

[58] Jung J.Y., Kim J.W., Suh C.H., Kim H.A. (2020). Roles of interactions between Toll-like receptors and their endogenous ligands in the pathogenesis of systemic juvenile idiopathic arthritis and adult-onset Still’s disease. Front. Immunol..

[59] Holzinger D., Foell D., Kessel C. (2018). The role of S100 proteins in the pathogenesis and monitoring of autoinflammatory diseases. Mol. Cell Pediatr..

[60] Bobek D., Grcevic D., Kovacic N., Lukic I.K., Jelusic M. (2014). The presence of high mobility group box-1 and soluble receptor for advanced glycation end-products in juvenile idiopathic arthritis and juvenile systemic lupus erythematosus. Pediatr. Rheumatol. Online J..

[61] Cantarini L., Giani T., Fioravanti A., Iacoponi F., Simonini G., Pagnini I., Spreafico A., Chellini F., Galeazzi M., Cimaz R. (2012). Serum amyloid A circulating levels and disease activity in patients with juvenile idiopathic arthritis. Yonsei Med. J..

[62] Chames P., Van Regenmortel M., Weiss E., Baty D. (2009). Therapeutic antibodies: Successes, limitations and hopes for the future. Br. J. Pharmacol..

[63] Flo T.H., Halaas O., Lien E., Ryan L., Teti G., Golenbock D.T., Sundan A., Espevik T. (2009). Human Toll-like receptor 2 mediates monocyte activation by Listeria monocytogenes, but not by group B streptococci or lipopolysaccharide. J. Immunol..

[64] Spiller S., Elson G., Ferstl R., Dreher S., Mueller T., Freudenberg M., Daubeuf B., Wagner H., Kirschning C.J. (2008). TLR4-induced IFN-gamma production increases TLR2 sensitivity and drives Gram-negative sepsis in mice. J. Exp. Med..

[65] Reilly M., Miller R.M., Thomson M.H., Patris V., Ryle P., McLoughlin L., Mutch P., Gilboy P., Miller C., Broekema M. (2013). Randomized, double-blind, placebo-controlled, dose-escalating phase I, healthy subjects study of intravenous OPN-305, a humanized anti-TLR2 antibody. Clin. Pharmacol. Ther..

[66] Philippe L., Alsaleh G., Suffert G., Meyer A., Georgel P., Sibilia J., Wachsmann D., Pfeffer S. (2012). TLR2 expression is regulated by microRNA miR-19 in rheumatoid fibroblast-like synoviocytes. J. Immunol..

[67] Jiang C., Zhu W., Xu J., Wang B., Hou W., Zhang R., Zhong N., Ning Q., Han Y., Yu H. (2014). MicroRNA-26a negatively regulates Toll-like receptor 3 expression of rat macrophages and ameliorates pristane induced arthritis in rats. Arthritis Res. Ther..

[68] Achek A., Shah M., Seo J.Y., Kwon H.K., Gui X., Shin H.J., Cho E.Y., Lee B.S., Kim D.J., Lee S.H. (2019). Linear and rationally designed stapled peptides abrogate TLR4 pathway and relieve inflammatory symptoms in rheumatoid arthritis rat model. J. Med. Chem..

[69] Samarpita S., Kim J.Y., Rasool M.K., Kim K.S. (2020). Investigation of Toll-like receptor (TLR) 4 inhibitor TAK-242 as a new potential anti-rheumatoid arthritis drug. Arthritis Res. Ther..

[70] Hegewald A.B., Breitwieser K., Ottinger S.M., Mobarrez F., Korotkova M., Rethi B., Jakobsson P.J., Catrina A.I., Wahamaa H., Saul M.J. (2020). Extracellular miR-574-5p induces osteoclast differentiation via TLR 7/8 in rheumatoid arthritis. Front. Immunol..

[71] Liu J., Wang X., Wang S., Liu F. (2021). Therapeutic potential of non-coding RNAs and TLR signalling pathways in Rheumatoid arthritis. Curr. Pharm. Biotechnol..

[72] Zhao W., Li D., Su Y., Zhao H., Pang W., Sun Y., Wu S. (2019). MicroRNA-147 negatively regulates expression of Toll-like receptor-7 in rat macrophages and attenuates pristane induced rheumatoid arthritis in rats. Am. J. Transl. Res..

[73] Zhu F., Wang D., Jiang W., Bhagat L., Agrawal S. (2015). Sa1757 targeting innate immune receptors to treat inflammatory bowel disease: Preclinical activity of IMO-9200, an antagonist of TLRS 7, 8, and 9 in mouse models of colitis. Gastroenterology.

[74] Danto S.I., Shojaee N., Singh R.S.P., Li C., Gilbert S.A., Manukyan Z., Kilty I. (2019). Safety, tolerability, pharmacokinetics, and pharmacodynamics of PF-06650833, a selective interleukin-1 receptor-associated kinase 4 (IRAK4) inhibitor, in single and multiple ascending dose randomized phase 1 studies in healthy subjects. Arthritis Res. Ther..

[75] Mihara K., Almansa C., Smeets R.L., Loomans E.E., Dulos J., Vink P.M., Rooseboom M., Kreutzer H., Cavalcanti F., Boots A.M. (2008). A potent and selective p38 inhibitor protects against bone damage in murine collagen-induced arthritis: A comparison with neutralization of mouse TNFalpha. Br. J. Pharmacol..

[76] Tsuchiya A., Imai K., Asamitsu K., Waguri-Nagaya Y., Otsuka T., Okamoto T. (2010). Inhibition of inflammatory cytokine production from rheumatoid synovial fibroblasts by a novel IkappaB kinase inhibitor. J. Pharmacol. Exp. Ther..

[77] Toubi E., Rosner I., Rozenbaum M., Kessel A., Golan T.D. (2000). The benefit of combining hydroxychloroquine with quinacrine in the treatment of SLE patients. Lupus.

[78] Barrat F.J., Meeker T., Chan J.H., Guiducci C., Coffman R.L. (2007). Treatment of lupus-prone mice with a dual inhibitor of TLR7 and TLR9 leads to reduction of autoantibody production and amelioration of disease symptoms. Eur. J. Immunol..

[79] Ostrach J. Dynavax Regains Full Rights to Investigational TLR 7/9 Inhibitor DV1179 Following Expiration of Collaboration with GSK. https://investors.dynavax.com/news-releases/news-release-details/dynavax-regains-full-rights-investigational-tlr-79-inhibitor.

[80] Zhu F.G., Jiang W., Bhagat L., Wang D., Yu D., Tang J.X., Kandimalla E.R., La Monica N., Agrawal S. (2013). A novel antagonist of Toll-like receptors 7, 8 and 9 suppresses lupus disease-associated parameters in NZBW/F1 mice. Autoimmunity.

[81] Mussari C.P., Dodd D.S., Sreekantha R.K., Pasunoori L., Wan H., Posy S.L., Critton D., Ruepp S., Subramanian M., Watson A. (2020). Discovery of potent and orally bioavailable small molecule antagonists of Toll-like receptors 7/8/9 (TLR7/8/9). ACS Med. Chem. Lett..

[82] Coley Pharmaceuticals (2009). First Safety Study in Humans of a Single Dose of CPG 52364. https://clinicaltrials.gov/ct2/show/NCT00547014.2009.

[83] Zheng C.Z., Shu Y.B., Luo Y.L., Luo J. (2017). The role of miR-146a in modulating TRAF6-induced inflammation during lupus nephritis. Eur. Rev. Med. Pharmacol. Sci..

[84] Kessel C., Holzinger D., Foell D. (2013). Phagocyte-derived S100 proteins in autoinflammation: Putative role in pathogenesis and usefulness as biomarkers. Clin. Immunol..

[85] Jung J.Y., Suh C.H., Sohn S., Nam J.Y., Kim H.A. (2016). Elevated high-mobility group B1 levels in active adult-onset Still’s disease associated with systemic score and skin rash. Clin. Rheumatol..

[86] Fukui R., Murakami Y., Miyake K. (2018). New application of anti-TLR monoclonal antibodies: Detection, inhibition and protection. Inflamm. Regen..

[87] Kanno A., Tanimura N., Ishizaki M., Ohko K., Motoi Y., Onji M., Fukui R., Shimozato T., Yamamoto K., Shibata T. (2015). Targeting cell surface TLR7 for therapeutic intervention in autoimmune diseases. Nat. Commun..

[88] Pawar R.D., Ramanjaneyulu A., Kulkarni O.P., Lech M., Segerer S., Anders H.J. (2007). Inhibition of Toll-like receptor-7 (TLR-7) or TLR-7 plus TLR-9 attenuates glomerulonephritis and lung injury in experimental lupus. J. Am. Soc. Nephrol..

[89] Duramad O., Fearon K.L., Chang B., Chan J.H., Gregorio J., Coffman R.L., Barrat F.J. (2005). Inhibitors of TLR-9 act on multiple cell subsets in mouse and man in vitro and prevent death in vivo from systemic inflammation. J. Immunol..

[90] Balak D.M., van Doorn M.B., Arbeit R.D., Rijneveld R., Klaassen E., Sullivan T., Brevard J., Thio H.B., Prens E.P., Burggraaf J. (2017). IMO-8400, a Toll-like receptor 7, 8, and 9 antagonist, demonstrates clinical activity in a phase 2a, randomized, placebo-controlled trial in patients with moderate-to-severe plaque psoriasis. Clin. Immunol..

[91] Barrat F.J., Meeker T., Gregorio J., Chan J.H., Uematsu S., Akira S., Chang B., Duramad O., Coffman R.L. (2005). Nucleic acids of mammalian origin can act as endogenous ligands for Toll-like receptors and may promote systemic lupus erythematosus. J. Exp. Med..

[92] Rommler F., Hammel M., Waldhuber A., Muller T., Jurk M., Uhlmann E., Wagner H., Vollmer J., Miethke T. (2015). Guanine-modified inhibitory oligonucleotides efficiently impair TLR7- and TLR9-mediated immune responses of human immune cell. PLoS ONE.

[93] Fletcher S., Hamilton A.D. (2006). Targeting protein-protein interactions by rational design: Mimicry of protein surfaces. J. R. Soc. Interface.

[94] Ebner S., Trieb M., Schonfeld M., Wietzorrek G., Santos-Sierra S. (2018). Decoy peptides derived from the extracellular domain of Toll-like receptor 2 (TLR2) show anti-inflammatory properties. Bioorg. Med. Chem..

[95] Horng T., Barton G.M., Medzhitov R. (2001). TIRAP: An adapter molecule in the toll signaling pathway. Nat. Immunol..

[96] Loiarro M., Sette C., Gallo G., Ciacci A., Fanto N., Mastroianni D., Carminati P., Ruggiero V. (2005). Peptide-mediated interference of TIR domain dimerization in MyD88 inhibits interleukin-1-dependent activation of NF-κB. J. Biol. Chem..

[97] Reddick L.E., Alto N.M. (2014). Bacteria fighting back: How pathogens target and subvert the host innate immune system. Mol. Cell.

[98] McCoy S.L., Kurtz S.E., Macarthur C.J., Trune D.R., Hefeneider S.H. (2005). Identification of a peptide derived from vaccinia virus A52R protein that inhibits cytokine secretion in response to TLR-dependent signaling and reduces in vivo bacterial-induced inflammation. J. Immunol..

[99] Tsung A., McCoy S.L., Klune J.R., Geller D.A., Billiar T.R., Hefeneider S.H. (2007). A novel inhibitory peptide of Toll-like receptor signaling limits lipopolysaccharide-induced production of inflammatory mediators and enhances survival in mice. Shock.

[100] Shukla N.M., Malladi S.S., Day V., David S.A. (2011). Preliminary evaluation of a 3H imidazoquinoline library as dual TLR7/TLR8 antagonists. Bioorg. Med. Chem..

[101] Wu Y., He S., Bai B., Zhang L., Xue L., Lin Z., Yang X., Zhu F., He P., Tang W. (2016). Therapeutic effects of the artemisinin analog SM934 on lupus-prone MRL/lpr mice via inhibition of TLR-triggered B-cell activation and plasma cell formation. Cell Mol. Immunol.

[102] Lamphier M., Zheng W., Latz E., Spyvee M., Hansen H., Rose J., Genest M., Yang H., Shaffer C., Zhao Y. (2014). Novel small molecule inhibitors of TLR7 and TLR9: Mechanism of action and efficacy in vivo. Mol. Pharmacol..

[103] Zhang S., Hu Z., Tanji H., Jiang S., Das N., Li J., Sakaniwa K., Jin J., Bian Y., Ohto U. (2018). Small-molecule inhibition of TLR8 through stabilization of its resting state. Nat. Chem. Biol..

[104] Bou Karroum N., Moarbess G., Guichou J.F., Bonnet P.A., Patinote C., Bouharoun-Tayoun H., Chamat S., Cuq P., Diab-Assaf M., Kassab I. (2019). Novel and selective TLR7 antagonists among the Imidazo[1,2-*a*]pyrazines, Imidazo[1,5-*a*]quinoxalines, and Pyrazolo[1,5-*a*]quinoxalines series. J. Med. Chem..

[105] Murgueitio M.S., Henneke P., Glossmann H., Santos-Sierra S., Wolber G. (2014). Prospective virtual screening in a sparse data scenario: Design of small-molecule TLR2 antagonists. Chem. Med. Chem..

[106] Wietzorrek G., Drexel M., Trieb M., Santos-Sierra S. (2019). Anti-inflammatory activity of small-molecule antagonists of Toll-like receptor 2 (TLR2) in mice. Immunobiology.

[107] Cheng K., Wang X., Zhang S., Yin H. (2012). Discovery of small-molecule inhibitors of the TLR1/TLR2 complex. Angew. Chem. Int. Ed. Engl..

[108] Cen X., Zhu G., Yang J., Yang J., Guo J., Jin J., Nandakumar K.S., Yang W., Yin H., Liu S. (2019). TLR1/2 specific small-molecule agonist suppresses leukemia cancer cell growth by stimulating cytotoxic T lymphocytes. Adv. Sci..

[109] Chavez S.A., Martinko A.J., Lau C., Pham M.N., Cheng K., Bevan D.E., Mollnes T.E., Yin H. (2011). Development of beta-amino alcohol derivatives that inhibit Toll-like receptor 4 mediated inflammatory response as potential antiseptics. J. Med. Chem..

[110] Isnardi I., Ng Y.S., Srdanovic I., Motaghedi R., Rudchenko S., von Bernuth H., Zhang S.Y., Puel A., Jouanguy E., Picard C. (2008). IRAK-4- and MyD88-dependent pathways are essential for the removal of developing autoreactive B cells in humans. Immunity.

[111] Loiarro M., Capolunghi F., Fanto N., Gallo G., Campo S., Arseni B., Carsetti R., Carminati P., De Santis R., Ruggiero V. (2007). Pivotal advance: Inhibition of MyD88 dimerization and recruitment of IRAK1 and IRAK4 by a novel peptidomimetic compound. J. Leukoc. Biol..

[112] Powers J.P., Li S., Jaen J.C., Liu J., Walker N.P., Wang Z., Wesche H. (2006). Discovery and initial SAR of inhibitors of interleukin-1 receptor-associated kinase-4. Bioorg. Med. Chem. Lett..

[113] Chiang E.Y., Yu X., Grogan J.L. (2011). Immune complex-mediated cell activation from systemic lupus erythematosus and rheumatoid arthritis patients elaborate different requirements for IRAK1/4 kinase activity across human cell types. J. Immunol..

[114] Hammaker D.R., Boyle D.L., Chabaud-Riou M., Firestein G.S. (2004). Regulation of c-Jun N-terminal kinase by MEKK-2 and mitogen-activated protein kinase kinase kinases in rheumatoid arthritis. J. Immunol..

[115] Clark A.R., Dean J.L. (2012). The p38 MAPK pathway in rheumatoid arthritis: A sideways look. Open Rheumatol. J..

[116] McIntyre K.W., Shuster D.J., Gillooly K.M., Dambach D.M., Pattoli M.A., Lu P., Zhou X.D., Qiu Y., Zusi F.C., Burke J.R. (2003). A highly selective inhibitor of I kappa B kinase, BMS-345541, blocks both joint inflammation and destruction in collagen-induced arthritis in mice. Arthritis Rheum..

[117] Paul A., Edwards J., Pepper C., Mackay S. (2018). Inhibitory-kappaB Kinase (IKK) alpha and nuclear factor-kappaB (NFkappaB)-Inducing Kinase (NIK) as anti-cancer drug targets. Cells.

[118] Drexel M., Kirchmair J., Santos-Sierra S. (2019). INH14, a small-molecule urea derivative, inhibits the IKKalpha/beta-dependent TLR inflammatory response. Chem. Bio. Chem..

[119] Manju S.L., Ethiraj K.R., Elias G. (2018). Safer anti-inflammatory therapy through dual COX-2/5-LOX inhibitors: A structure-based approach. Eur. J. Pharm. Sci..

[120] Roome T., Aziz S., Razzak A., Aslam Z., Lubna Jamali K.S., Sikandar B., Fatima T., Abidi L., Imran M., Faizi S. (2019). Opuntioside, opuntiol and its metallic nanoparticles attenuate adjuvant-induced arthritis: Novel suppressors of Toll-like receptors -2 and -4. Biomed. Pharmacother..

[121] Arenas-Padilla M., Mata-Haro V. (2018). Regulation of TLR signaling pathways by microRNAs: Implications in inflammatory diseases. Cent. Eur. J. Immunol..

[122] Bartel D.P. (2009). MicroRNAs: Target recognition and regulatory functions. Cell.

[123] Tang B., Xiao B., Liu Z., Li N., Zhu E.D., Li B.S., Xie Q.H., Zhuang Y., Zou Q.M., Mao X.H. (2010). Identification of MyD88 as a novel target of miR-155, involved in negative regulation of Helicobacter pylori-induced inflammation. FEBS Lett..

[124] Wei J., Huang X., Zhang Z., Jia W., Zhao Z., Zhang Y., Liu X., Xu G. (2013). MyD88 as a target of microRNA-203 in regulation of lipopolysaccharide or Bacille Calmette-Guerin induced inflammatory response of macrophage RAW264.7 cells. Mol. Immunol..

[125] Xu G., Zhang Z., Xing Y., Wei J., Ge Z., Liu X., Zhang Y., Huang X. (2014). MicroRNA-149 negatively regulates TLR-triggered inflammatory response in macrophages by targeting MyD88. J. Cell Biochem..

[126] Jiang R., Zhang H., Zhou J., Wang J., Xu Y., Zhang H., Gu Y., Fu F., Shen Y., Zhang G. (2021). Inhibition of long non-coding RNA XIST upregulates microRNA-149-3p to repress ovarian cancer cell progression. Cell Death Dis..

[127] Zhu S., Pan W., Song X., Liu Y., Shao X., Tang Y., Liang D., He D., Wang H., Liu W. (2012). The microRNA miR-23b suppresses IL-17-associated autoimmune inflammation by targeting TAB2, TAB3 and IKK-alpha. Nat. Med..

[128] Sujitha S., Rasool M. (2017). MicroRNAs and bioactive compounds on TLR/MAPK signaling in rheumatoid arthritis. Clin. Chim. Acta.

[129] Olivieri F., Rippo M.R., Prattichizzo F., Babini L., Graciotti L., Recchioni R., Procopio A.D. (2013). Toll like receptor signaling in “inflammaging”: MicroRNA as new players. Immun. Ageing.

[130] Kuznik A., Bencina M., Svajger U., Jeras M., Rozman B., Jerala R. (2011). Mechanism of endosomal TLR inhibition by antimalarial drugs and imidazoquinolines. J. Immunol..

